# The Association Among Achievement Goal Orientations, Academic Performance, and Academic Well-Being Among Chinese Medical Students: A Cross-Sectional Study

**DOI:** 10.3389/fpsyg.2021.694019

**Published:** 2021-08-02

**Authors:** Qing-lin Li, Ji-yang Zhao, Jing Tian, Tao Sun, Chen-xi Zhao, Hai-chen Guo, Li-yan Zhu, Rui Gao, Li-bin Yang, De-pin Cao, Shu-e Zhang

**Affiliations:** ^1^Department of Health Management, School of Health Management, Harbin Medical University, Harbin, China; ^2^Department of Health Management to School of Medicine, Hang Zhou Normal University, Hangzhou, China

**Keywords:** association, academic well-being, academic performance, achievement goal orientations, medical students

## Abstract

Learning motivation is a significant factor that ensures quality in medical education, and might affect the academic performance and well-being of medical students. This study aimed to explore the status of achievement goal orientations among medical students in China and to further identify the association among academic performance, academic well-being, and achievement goal orientations. Data were collected through a cross-sectional, anonymous survey conducted with 3,511 respondents (effective response rate = 81.7%), from four medical universities in China, and demographic factors, achievement goal orientations, academic performance, and academic well-being were assessed. The average score of achievement goal orientations of Chinese medical students suggested a difference in demographic factors, including sex, year of study, experience of leadership cadre, and family income. Both mastery and performance-avoidance goals were associated with academic performance, subjective academic stress, subjective learning adaptability, subjective sleep quality, and subjective well-being. Performance-approach goals were related to academic performance, subjective academic stress, and subjective learning adaptability. The achievement goal orientations of the medical students in this study were at a middle level. The findings emphasize the importance of mastery goals for promoting the academic performance and well-being of medical students. More care and attention toward achievement goal orientations can be beneficial for the improvement of the academic performance and well-being among medical students.

## Introduction

Considered as motivators of human behavior, achievement goal orientations are defined as the subjective expression of individual pursuits (Nicholls, 1984), and are usually divided into three types— mastery goals (that is to develop ability), performance-approach goals (that is to prove ability), and performance-avoidance goals (that is to avoid incompetence; Elliot and Harackiewicz, 1996). Achievement goal orientations have been widely discussed by scholars in recent decades (Tuominen-Soini et al., 2008; Hall et al., 2015; King and Mendoza, 2020; Sideridis, 2020). A considerable body of literature suggests that achievement goal orientations play an important role in promoting academic development among students, and it can enhance their engagement in and enjoyment of studying. Furthermore, it is beneficial as it alleviates psychological stress and promotes the use of active learning strategies, thus leading to high academic achievement (Daniels et al., 2008; Tuominen-Soini et al., 2008, 2012; Gonçalves et al., 2017). Achievement goal orientations are also inseparable from some emotional experiences, such as stress, anxiety, depression, and burnout (Daniels et al., 2008; Tuominen-Soini et al., 2012; Gonçalves et al., 2017). Undoubtedly, continuous attention should be given to achievement goal orientations.

Since the concept of achievement goal orientations was proposed, there have been many studies on junior middle school, high school, and ordinary college students; however, only a few studies have focused on medical students (Giota, 2006; Tian et al., 2017; King and Mendoza, 2020; You, 2021). In recent years, the learning motivation of medical students in China has been one of the main practical concerns (Youhao et al., 2019). Due to the vast curriculum content and high pressure to learn, medical students are prone to negative tendencies such as academic dishonesty and cynicism (Ahmadi et al., 2009). In China, medical students face demanding learning tasks and long study periods, which can easily lead to academic stress and affect their learning engagement and emotional experience (Hong et al., 2010; Jiali et al., 2019; Lew et al., 2019). In addition, the outcome expectation can be attached to a greater importance in the Chinese cultural context, which may cause learning motivation problems for medical students and affect their achievement goal orientations (Liu and Tein, 2005). Some studies have shown that the achievement goal orientations of Chinese medical students are more negative than those of general students (Kim et al., 1997; Liu et al., 2000; Swetlik and Franco, 2018; Chen et al., 2020). Therefore, we believe that the level of achievement goal orientations of Chinese medical students is worrying and deserves close attention.

Low academic performance is a frequent obstacle in medical students’ careers as doctors, as it is a key index that reflects educational outcomes (Stinebrickner and Stinebrickner, 2007), which are closely related to students’ learning motivation. Medical students have diverse learning motivations and motivational orientations influenced by individual factors, family factors, and school factors (Korpershoek et al., 2015). Those with mastery goals attempt to understand the topic at hand, gain knowledge, and improve their skills, whereas students with performance-approach goals are focused on outperforming others (Korpershoek et al., 2015). However, Giota (2006) considered that performance-approach goals may be more often linked to students’ ability-related concerns such as anxiety and surface level strategy use, including lower levels of academic achievement. Research on Indian medical students showed that the work-avoidance type of goal orientation among the lower performing group may account for their lower performance scores when compared with the higher performing group (Barkur et al., 2013). They believed that medical students with performance-avoidance goals hide themselves as they try to avoid engaging in any activity. Meanwhile, other studies demonstrated that achievement goal orientations have no impact on the academic performance among medical students (Tapola et al., 2014; Korpershoek et al., 2015; Schwinger et al., 2016). Unlike Western countries, the achievement goal orientation of Chinese students is unique, dynamic, and uncertain due to the excessive attention to academic achievements from both families and individuals (Zang and Carrasquillo, 1995; Yang and Zhou, 2008). Therefore, it is worthwhile to further explore the relationship between academic performance and achievement goal orientations in China. In addition, some scholars concluded that achievement goals are related to emotions and cognitions that not only contribute to effective learning, but are also generally linked with well-being (Kaplan and Maehr, 1999; Tuominen-Soini et al., 2008).

In addition to performance, academic well-being is as important as academic success. Medical universities face the challenge of creating an academic environment that motivates students to engage in rigorous learning without compromising their health and well-being (Noddings, 2010). The recent years have seen a surge of recognition regarding the importance of students’ stress levels and emotional well-being (Noddings, 2010). Stress is a normal and healthy response to difficult negative learning events; however, some Chinese medical students excessively face negative emotional experiences such as depression or anxiety, and even develop suicidal tendencies (Jiali et al., 2019; Lew et al., 2019). Academic well-being is regarded as the emotional experience during students’ learning activities and it can be perceived as an evaluation of the entire learning process (Jing and Yu, 2015; Yuting et al., 2020). Studies have revealed that academic well-being is significantly associated with learning motivation (Tian et al., 2017; Tuominen et al., 2020). Tuominen-Soini et al. (2008) reported that learning motivation associated with self-improvement and growth was positively related to various indices of students’ well-being, whereas avoidance tendencies and concerns with demonstrating one’s competence were linked to different types of adjustment problems. They believed that some students have a stronger tendency to validate their competence, which makes them more vulnerable to situations that potentially imply incompetence or otherwise pose a threat to their self-esteem. They also thought that students with avoidance tendencies were challenged by avoidance, having low persistence in the face of difficulty, and negative self-cognition when confronting obstacles, whereas an opposite pattern was observed for mastery-oriented students. Research on students in Finland from lower and upper secondary school showed that those in the mastery-oriented group displayed the most adaptive pattern of motivation, academic achievement, and well-being (Tuominen et al., 2020). Thus far, it has been difficult for academic circles to determine the relationship between achievement goal orientations and academic well-being in different cultural settings due to different measuring tools; however, it is certain that the association between achievement goal orientations and academic well-being among medical students needs to be discussed using locally adapted measuring tools in China.

### Goal of Study

We conducted a survey on medical students to evaluate the following— (1) the status of achievement goal orientations and its influencing factors and (2) the association among achievement goal orientations, academic performance, and academic well-being.

## Materials and Methods

### Participants and Procedures

**FIGURE 1:**
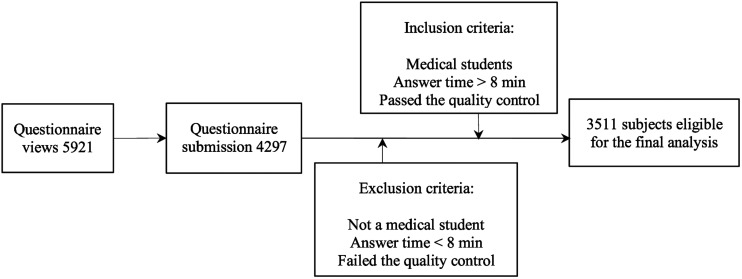
Flowchart of data acquisition.

Considering the time-effectiveness, cost-effectiveness and accessibility (Chang and Vowles, 2013), a cross-sectional anonymous online survey was conducted from May to June 2019 in current study. The multistage stratified convenient sampling method was used to collect data among medical students. Firstly, the procedures of this study were reviewed and approved by the Ethics Committee of the Institutional Review Board of Harbin Medical University (ECHMU). According to the calculation method and standard requirements of the cross-sectional sample size from Zhou et al. (2017), the minimum sample size of this study was calculated to be 1,824 participants. Considering that the minimum response rate is 50%, the sample size of this study should be expanded to at least 3,648 participants. In order to further ensure the data quality, we finally determined the number of respondents to be 5,000 participants. Secondly, we contacted 4 teachers in charge of student affairs and the academic administrators as the original deliverers of the survey. Prior to the formal release of the questionnaire, we trained the original deliverers of the survey. After understanding the content and purpose of the survey, the original deliverers recruited potential and qualified medical students from four regions, including Harbin, Jiamusi, Mudanjiang, and Qiqihar. In each region, one medical college was hierarchically selected, totaling four medical colleges. The characteristics of the medical colleges differed by size, academic programs, research performance, admission scores and number of medical students. In addition, different classes and grades were randomly selected in each university. Thirdly, the survey was conducted through the online survey platform ‘‘Questionnaire Star.’’ Once informed consent was obtained, a web page link to our questionnaire survey^1^ was sent to each participant via mobile phone during students’ spare time. Each participant is only allowed to reply once. The researchers monitored the collected questionnaires in real time through the platform of “Questionnaire Star” and used the platform to effectively manage the data. The senior investigators conduct quality control by checking the collected questionnaires daily. In the past, our team has successfully used this survey method to finish a series of studies (Zhang et al., 2018; Shi et al., 2021). The link was sent to 5,921 participants, and 4,297 questionnaires have been submitted successfully. Our final sample selection strictly adheres to exclusion criteria for data management and quality control. Finally, we collected 3,511 valid questionnaires with an effective rate of 81.7%, excluding incomplete answers, failed the quality control questions (such as how carefully you filled out the questionnaire) and questionnaires that took <8 min to answer (the minimum answering time was 8 min in the preliminary investigation). The inclusion criteria required the participants to be students at a medical college and to voluntarily and truthfully cooperate with the online questionnaire survey. The specific data acquisition process is showed in Figure 1.

### Study Instruments

A self-administered questionnaire consisting of demographic characteristics, achievement goal orientations, academic performance, and academic well-being was used, based on the objectives of this study.

### Demographic Characteristics

Information on five demographic characteristics was collected from a self-designed questionnaire— sex, students’ provenience, year of study, experience of leadership cadre, and family income. Students’ provenience was divided into two categories: “rural” and “urban.” The year of study was collected as a continuous variable, from 1 to 5. The leadership experience was divided into “student leaders” and “ordinary students.” Options for family income included “≤¥5,000,” “¥5,001–¥10,000,” “¥10,001–¥20,000,” and “≥¥20,001.”

### Achievement Goal Orientations

Achievement goal orientations were measured using the Chinese version of the Survey of Achievement Goal Orientations (SAGO; Elliot and Harackiewicz, 1996), which was translated by Wang et al. (Yanfei et al., 2011). The SAGO-13 consists of 13 items and three dimensions— mastery goals (four items), performance-approach goals (six items), and performance-avoidance goals (three items). Each item was scaled from 1 = *completely inconsistent* to 5 = *fully consistent*. In this study, the achievement goal orientation score was calculated by summing up the related questions ranging from 1 to 13. Higher values indicated higher levels of achievement goal orientation. Its reliability and validity have been tested among Chinese students by Wang (Dan, 2018). In this study, the Cronbach’s alpha coefficients of the SAGO-13 were 0.752 (mastery goals), 0.864 (performance-approach goals), and 0.507 (performance-avoidance goals).

### Academic Performance

The measurement of academic performance refers to the dimension of academic performance in the academic achievement scale developed by Wang et al. (Yanfei et al., 2011) which includes Chinese characteristics. The scale consists of four items— moral education, intellectual education, sports, and synthesis. The response format is a five-point Likert scale, ranging from 1 = *downstream level* to 5 = *upstream level*, where higher scores indicate higher academic performance. The Cronbach’s alpha for the scale used in this study was 0.874.

### Academic Well-Being

The term of well-being is commonly used but inconsistently defined in varied studies (Pollard and Lee, 2003). There are differences in the measurement dimensions of well-being among cross cultural backgrounds and characteristic subjects. The measurement of well-being is relatively diverse (Kim et al., 2016; Schütz et al., 2018; Strelhow et al., 2020). Combining the characteristics of academic well-being among medical students, we considered that a single item of subjective well-being cannot fully reflect the overall well-being of medical students. Thus, considering important aspects of the group characteristics among Chinese medical students, we included three items— sleep quality, academic stress, and learning adaptability, representing the physical, psychological, and social dimensions. The items were measured using a 10-point Likert-type scale, ranging from *very poor* to *perfect*. Previous studies have shown that the single item scales of academic stress (Azila-Gbettor et al., 2015), sleep quality (Werneck et al., 2020), subjective well-being (Ashenfelter and Rouse, 1998), and learning adaptability (Tjin et al., 2018) have consistent reliability and validity.

### Data Analysis

The participants’ demographic standardized regression coefficients were expressed by (β) and (*P*) in each step of the regression model. The characteristics were reported as sample information. Pearson’s correlation coefficients were calculated to estimate the correlations between achievement goal orientations, academic performance, and academic well-being. Descriptive statistics of the demographic and achievement goal orientations were indicated using the mean, standard deviation (SD), number (N), and percentage (%). Study variables were compared between sex groups, student source groups, year of study groups, experience of leadership cadres, and family monthly income groups by one-way ANOVA analyses. When the one-way ANOVAs were found to be significant, least-significant-difference tests (LSDs) were conducted for multiple comparisons. A multiple linear regression analysis was performed to examine the relationships between the variables. The Cronbach’s alpha coefficient was used to measure the reliability of the measuring tools that we used. All the statistical analyses were performed using the IBM SPSS Statistics 22.0, and a two-tailed *P* < 0.05 was considered statistically significant.

## Results

### Demographic Characteristics of Participants

The demographic characteristics of the participants included sex, provenience, year of study, experience of leadership cadre, and family monthly income. The percentage of participants who were female, urban, and student leaders were 66.0, 54.1, and 35.5%, respectively. Participants’ years of study were 1 (30.8%), 2 (28.5%), 3 (22.7%), 4 (6.7%), and 5 (11.3%). The family monthly income levels of 41.8% of the participants were below ¥5,000, 48.5% had this level between ¥5,001–¥10,000, 9.0% had between ¥10,001–¥20,000, and 0.7% had a family income level above ¥20,001.

### The Score of Achievement Goal Orientations Among Medical Students

The results indicated that the achievement goal orientations (*M* = 3.57, *SD* = 0.58) among medical students were at middle level. Other scores of achievement goal orientations, from highest to lowest included mastery goals (*M* = 3.85, *SD* = 0.66), performance-avoidance goals (*M* = 3.48, *SD* = 0.74), and performance-approach goals (*M* = 3.42, *SD* = 0.80), as presented in Table 1.

**TABLE 1:** The Means, standard deviations (SD) score of achievement goal orientations among medical students (*n* = 3511).

Variable	*M* ± *SD*	Min-Max
Achievement goal orientations	3.57 ± 0.58	1–5
Mastery goals	3.85 ± 0.66	1–5
Performance-approach goals	3.42 ± 0.80	1–5
Performance- avoidance goals	3.48 ± 0.74	1–5

### The Difference Between Participants’ Characteristics and Multiple Variables Scores

There was a significant difference in the scores on achievement goal orientations depending on students’ demographics, including sex, year of study, experience of leadership cadre, and family income. A further pairwise comparison using the LSD method showed significantly different scores for mastery goals, performance-approach goals, performance-avoidance goals, and achievement goal orientations in year of study and family monthly income among medical students. The descriptive association between respondents’ characteristics and mastery goals, performance-approach goals, performance-avoidance goals, and achievement goal orientation scores can be seen in Table 2.

**TABLE 2:** One-way ANOVO analysis of mastery goals, performance-approach goals, performance- avoidance goals, and achievement goal orientations of medical students (*n* = 3511).

Variable		*n*	%	Mastery goals*M*±*SD*	Performance-approach goals*M*±*SD*	Performance-avoidance goals*M*±*SD*	Achievement goal orientations *M*±*SD*
Sex	Male	1,194	34.0	3.90 ± 0.69	3.46 ± 0.83	3.47 ± 0.79	3.60 ± 0.62
	Female	2,317	66.0	3.82 ± 0.65	3.40 ± 0.78	3.49 ± 0.71	3.55 ± 0.56
	*t*			3.476	2.072	-0.803	2.290
	*P*			0.001	0.038	0.422	0.022
Student source	Rural	1,612	45.9	3.84 ± 0.64	3.41 ± 0.77	3.48 ± 0.73	3.56 ± 0.57
	Urban	1,899	54.1	3.85 ± 0.68	3.43 ± 0.82	3.48 ± 0.75	3.57 ± 0.60
	*t*			-0.290	-0.503	-0.314	-0.512
	*P*			0.772	0.615	0.753	0.609
Year of study	➀One	1,082	30.8	3.90 ± 0.64	3.53 ± 0.73	3.60 ± 0.70	3.66 ± 0.54
	➁Two	1,001	28.5	3.86 ± 0.67	3.44 ± 0.82	3.45 ± 0.75	3.57 ± 0.59
	➂Three	796	22.7	3.77 ± 0.69	3.30 ± 0.83	3.40 ± 0.76	3.47 ± 0.61
	➃Four	235	6.7	3.81 ± 0.69	3.34 ± 0.80	3.35 ± 0.75	3.49 ± 0.59
	➄Five	397	11.3	3.85 ± 0.63	3.36 ± 0.81	3.48 ± 0.73	3.54 ± 0.57
	*F*			4.848	11.331	11.536	14.549
	*P*			0.001	0	0	0
*LSD*				➀>➁>➂	➀>➁>➂, ➀>➃, ➀>➄	➀>➄>➃, ➀>➁, ➀>➂	➀>➁>➂, ➀>➃, ➀>➄, ➄>➂
Experience of leadership cadre	Student leaders	1,245	35.5	3.94 ± 0.65	3.59 ± 0.77	3.46 ± 0.77	3.67 ± 0.59
	Ordinary students	2,266	64.5	3.80 ± 0.66	3.33 ± 0.79	3.49 ± 0.73	3.51 ± 0.57
	*t*			6.196	9.179	-1.438	7.527
	*P*			0	0	0.150	0
Family income (RMB)	➀ = 5,000	1,468	41.8	3.83 ± 0.65	3.39 ± 0.79	3.49 ± 0.74	3.55 ± 0.58
	➁5,001–10,000	1,701	48.5	3.84 ± 0.66	3.43 ± 0.79	3.47 ± 0.74	3.57 ± 0.57
	➂10,001–20,000	316	9.0	3.94 ± 0.71	3.50 ± 0.85	3.49 ± 0.76	3.63 ± 0.65
	➃ = 20,001	26	0.7	4.11 ± 0.73	3.81 ± 0.88	3.51 ± 0.83	3.83 ± 0.65
	*F*			3.928	4.268	0.179	3.822
	*P*			0.008	0.005	0.911	0.010
*LSD*				➃>➀, ➂>➀, ➃>➁, ➂>➁	➃>➀, ➂>➀, ➃>➁		➃>➀, ➂>➀, ➃>➁

### Multiple Linear Regression Analysis Models

All the variables were significantly correlated with each other. Achievement goal orientations were positively related to academic performance (*r* = 0.312, *P* < 0.01) and academic well-being (*r* = 0.147, *P* < 0.01). Therefore, a multiple linear regression analysis was performed to evaluate the influence of academic performance and well-being on the achievement goal orientations of medical students after eliminating the effects of the demographic variables. The results showed that mastery goals of medical students were positively associated with their academic performance (β = 0.199, *P* < 0.01), subjective learning adaptability (β = 0.261, *P* < 0.01), subjective sleep quality (β = 0.090, *P* < 0.01), and subjective well-being (β = 0.178, *P* < 0.01), and negatively related to their subjective academic stress (β = −0.120, *P* < 0.01). The performance-approach goals were positively correlated with academic performance (β = 0.267, *P* < 0.01) and subjective academic stress (β = 0.118, *P* < 0.01), and negatively associated with their subjective learning adaptability (β = −0.040, *P* < 0.05). The performance-avoidance goals were positively related to students’ subjective academic stress (β = 0.107, *P* < 0.01), and negatively correlated with their academic performance (β = −0.148, *P* < 0.01), subjective learning adaptability (β = −0.057, *P* < 0.01), subjective sleep quality (β = −0.051, *P* < 0.01), and subjective well-being (β = −0.051, *P* < 0.01), as shown in Table 3.

**TABLE 3:** Regression analysis of achievement goal orientations to academic performance and academic well- being among medical students.

Variable	Academic performance	Academic well-being			
		Subjective academic stress	Subjective learning adaptability	Subjective sleep quality	Subjective well-being
Control variables					
Sex	0.070	–0.083	–0.002	0.096	0.056
Year of study	0.050	0.206	0.043	–0.002	–0.041
Student source	–0.017	0.013	0.017	0.045	0.019
Experience of leadership cadre	–0.186	–0.037	–0.107	–0.008	–0.046
Family income	0.032	–0.044	0.034	0.088	0.030
Predictor variables					
Mastery goals	0.199**	−0.120**	0.261**	0.090**	0.178**
Performance-approach goals	0.267**	0.118**	−0.040*	–0.004	–0.030
Performance- avoidance goals	−0.148**	0.107**	−0.057**	−0.051**	−0.051**
*F*	112.529**	37.752**	38.654**	13.130**	17.446**
*R* ^2^	0.204**	0.079**	0.081**	0.029**	0.038**
*ΔR^2^*	0.138**	0.033**	0.060**	0.009**	0.029**

Note: *p < 0.05, **p < 0.01.

## Discussion

### Current Status of Achievement Goal Orientations Among Medical Students

This study investigated the status of achievement goal orientations among Chinese medical students. Our results showed that the mean score of achievement goal orientations among the participating medical students was 3.57 ± 0.58 (mean ± *SD*). The mastery goal score was the highest and the performance-approach goal score was the lowest. The findings suggested that the achievement goal orientations of Chinese medical students were at a middle level, which was consistent with the scores reported by Niu (Weina, 2019), and should be of great concern. In fact, due to the particularities of the medical discipline, medical students tend to enhance their ability improvement (Xue et al., 2018). Moreover, medical students’ mistakes occurring during clinical practice might threaten patients’ safety and health, therefore, avoiding failure has become a common achievement goal orientation among medical students.

### The Difference in Achievement Goal Orientations Across Various Socio-Demographic Factors

Among demographic factors, sex, year of study, experience of leadership cadre, and family income were found to have an impact on the achievement goal orientations of medical students. There are differences in the social roles between men and women, as men are more affected by perceived social pressure. Moreover, to maintain their self-realization and independence (Hui et al., 2018; Xiaozhou et al., 2019), men are likely to show positive goal orientations. Furthermore, compared with students from other disciplines or the general population, medical students have higher admission scores for comprehensive quality and study enthusiasm before joining a medical university, which might contribute to the higher scores of achievement goal orientations. After entering the second university year, medical students have gradually adapted to university life. They pursue comprehensive ability development rather than a single achievement goal orientation during this learning stage. Once they encounter the internship stage in the third year, medical students face the requirements of heavy clinical skill operations and increased learning tasks. A learning environment with excessive stress and workload is likely to lead to negative emotions, which may decrease the level of mastery and performance-approach goals, and can even impair their mental health. Medical students in the fifth year experience various types of conflict stress resulting from the postgraduate entrance examination, medical practitioners examination, career choices, and graduating practices. During this stage, they tend to avoid failure in the examinations mentioned and make more efforts to learn, thus the performance-avoidance goals slightly increase. In fact, medical school teachers should provide different learning strategies for students of different sexes and years of study. As a previous study presented (Xue, 2019), medical students serving as leadership cadres need to organize daily activities, and they have higher abilities, responsibility, confidence, and willpower. Therefore, when facing difficulties and setbacks, they have higher endurance and resilience levels (Yanfei et al., 2011). Administrators should provide more opportunities for medical students to obtain the experience of leadership cadres, thereby helping more medical students to establish positive goals (King and Mendoza, 2020). In addition, medical students with low family income may experience more stress and anxiety in their studies and their lives. It is inevitable that their learning process is hindered by insufficient investment; thus, the scores of mastery and performance-approach goals are correspondingly lower. Medical students with family economic difficulties should be provided additional economic and psychological support. Understanding the demographic factors of achievement goal orientation provides a comprehensive model for interventions and policies aimed to improve the achievement goal orientations of medical students.

### The Association Between Achievement Goal Orientations and Academic Performance Among Medical Students

The results also revealed that medical students’ mastery and performance-approach goals were positively associated with academic performance, while performance-avoidance goals were negatively related to academic performance, similarly to the findings of a previous study on university students in the United States (Alhadabi and Karpinski, 2019). As previous studies presented, medical students with mastery goals exhibit positive self-perception and adaptive behaviors (such as enthusiasm, persistence, interest, and effective learning strategies; Giota, 2006; Chan et al., 2012; King et al., 2012); thus, excellent academic performance can be achieved by them. According to the matching hypothesis proposed by Harackiewicz and Sansone (1991), goal effects depend on the general context in which goals are pursued (Barron and Harackiewicz, 2001). Parents and teachers who pay excessive attention to educational outcomes, in the Chinese context, might stimulate an increase in performance-approach goals and increase students’ motivation to achieve higher performance levels. Medical students with a performance-approach goal orientation tend to focus on how to achieve better outcomes. This type of goal often allows the acquisition of skills in order to try to outdo others, to prove ability and superiority, and to pursue tasks with the intent of gaining a positive evaluation from others. Therefore, students with performance-approach goals may have a good academic performance. Some scholars have confirmed that performance-avoidance goals are positively associated with heavy burnout, negative learning attitudes, and low learning enthusiasm (Tuominen-Soini et al., 2012). Medical students with performance-avoidance goals also showed the most unsuitable pattern in academic performance and lower participation in university activities (Tuominen-Soini et al., 2008, 2012; Francisco et al., 2016). Moreover, medical students with avoidance tendencies exhibit negative self-perceptions and attitudes, effort withdrawal, and self-handicapping (SkaalviK, 1997). Thus, it can be said that students with performance-avoidance goals tend to have worse academic performance levels (Tapola and Niemivirta, 2008). As a result, mastery and performance-approach goals should be nurtured to improve the academic performance of medical students. However, there is a study showed that the COVID-2019 pandemic blockade has affected academic performance with varying degrees (Mahdy, 2020). But there is no relevant research on achievement goal orientations in this period. It is not possible to rule out whether the students’ status of achievement goal orientations were influenced by their academic performance. Therefore, to foster positive achievement goal orientations in medical students, it is important to enhance their academic performance through problem-based learning, case-based learning, self-directed learning, and conducting continuous assessments (Barkur et al., 2013).

### The Association Between Achievement Goal Orientations and Academic Well-Being Among Medical Students

In this study, medical students’ mastery goals were negatively associated with academic stress and positively related to learning adaptability, sleep quality, and subjective well-being. Surprisingly, medical students with performance-approach goals were positively correlated with academic stress and negatively associated with learning adaptability. Medical students’ performance-avoidance goals were positively correlated with academic stress and negatively related to learning adaptability, sleep quality, and subjective well-being. These results were inconsistent with a previous study on adolescents in public middle schools in China (Tian et al., 2017), but were similarly to the findings of a previous study on students in compulsory school and upper-secondary school in Swedish (Bergh and Giota, 2020). The reasons for these discrepancies may be the differences in cultural backgrounds and research objects. In China, teachers and parents attached great importance to the education of students (Liu and Tein, 2005). Compared with other types of students, medical students are faced with more learning tasks (Jiali et al., 2019; Lew et al., 2019). Medical students with mastery goals have positive self-perceptions and participation consciousness (Tuominen-Soini et al., 2008, 2012; Gonçalves et al., 2017; Tuominen et al., 2020). Moreover, mastery goals are correlated with various positive and adaptive patterns of coping and affect (Daniels et al., 2008). Therefore, these students may experience less academic stress while having higher learning adaptability, sleep quality, and subjective well-being; however, medical students with performance-approach goals may pay too much attention to the goal and experience negative emotions such as anxiety, pressure, fear, and burnout (Tuominen-Soini et al., 2011; Zhang et al., 2016). This may lead to academic stress and a decline in learning adaptability. Those who adopt performance-avoidance goals are often correlated with negative emotional experiences and maladaptive outcomes such as stress, anxiety, hopelessness, and shame (SideridiS, 2005; Pekrun et al., 2006; Tapola and Niemivirta, 2008; Tuominen-Soini et al., 2008, 2012; Luo et al., 2011; Francisco et al., 2016). Hence, their academic stress is high, while the learning adaptability, sleep quality, and subjective well-being are low. It is likely that medical students with performance-approach goals who are exceedingly concerned with surpassing others and succeeding in school present negative cognition and emotion when faced with study difficulties, which might pose a further threat to academic well-being (Grant and Dweck, 2003). Medical students with performance-approach goals obtained better academic results more easily, even with a low level of academic well-being. Unfortunately, studies have shown that the pandemic of COVID-2019 had a significant impact on well-being among students (Li et al., 2020; Marques et al., 2021). But there is no relevant research on achievement goal orientations in this period. It is not possible to rule out whether they have a specific achievement goal orientation due to their status of academic well-being. In addition to caring for the academic performance of medical students, more attention needs to be paid to academic well-being. The significant association between mastery goals and academic well-being should be taken into consideration when formulating solutions to increase the latter. Therefore, we should create a harmonious competitive environment for medical students to improve their academic well-being and performance by boosting positive achievement goal orientations (Eccles and Midgley, 1989).

## Limitations

Although there are valuable discoveries, the present study has several limitations. First, a convenient sample was used as we recruited participants from four regions in the same province in China, which is very small compared to the Chinese medical student population and may limit the generalizability of the findings for other regions. Second, its cross-sectional nature prevented the establishment of a causal relationship between the variables. Therefore, an important suggestion is that similar longitudinal studies should be conducted in the future. Third, the choice to measure academic stress, learning adaptability, sleep quality, and subjective well-being using single items weakens the assessment and shortens the validity of the measurement tools. Besides, although a series of quality control measures were taken when we collected the data, the unsure deviations may exist caused by the online cross-sectional survey. Therefore, a rigorous sampling technique and a larger sample size are needed for a future study.

## Conclusion

In summary, this study found that the achievement goal orientations of Chinese medical students are at a middle level. Mastery goals are positively associated with academic performance and well-being. Chinese medical students with performance-approach goals were positively related to academic performance and negatively correlated with academic well-being. Those with performance-avoidance goals were negatively associated with academic performance and well-being. Accordingly, medical students should be encouraged to achieve their mastery goals. In addition, medical students should be provided with more psychological and social support and be helped throughout their learning careers. Lastly, academic well-being interventions should be carried out to enhance the achievement goal orientations of the Chinese medical students.

## Data Availability Statement

The raw data supporting the conclusions of this article will be made available by the authors, without undue reservation.

## Ethics Statement

The study was reviewed and approved by the Ethics Committee of the Institutional Review Board of Harbin Medical University (ECHMU). Due to the online survey approach, the written informed consent could not be received. Therefore, verbal informed consent for survey was approved by the ECHMU and obtained from each participate.

## Author Contributions

All authors made substantial contributions to the whole study. Q-lL and J-yZ came up with the idea and designed the study with the help of D-pC. JT and TS done the acquisition of data with help from C-xZ and H-cG. S-eZ entered the data into SPSS with the help from RG. Q-lL analyzed and interpreted the data with the help from L-yZ. S-eZ and L-bY conducted the focus group discussion. All authors contributed in preparation and submission of manuscript and, read and approved the final manuscript.

## Conflict of Interest

The authors declare that the research was conducted in the absence of any commercial or financial relationships that could be construed as a potential conflict of interest.

## Publisher’s Note

All claims expressed in this article are solely those of the authors and do not necessarily represent those of their affiliated organizations, or those of the publisher, the editors and the reviewers. Any product that may be evaluated in this article, or claim that may be made by its manufacturer, is not guaranteed or endorsed by the publisher.

## Footnotes

1
http://www.wenjuan.com/

## References

[B1] Ahmadi K. Fathi-Ashtiani A. Ghaffari A. Hossein-Abadi F. H. (2009). Medical Students’ Educational Adjustment and Motivation Power in Compare With Other Academic Majors: a Prospective Study. *J. Appl. Sci.* 9 1350–1355. 10.3923/jas.2009.1350.1355

[B2] Alhadabi A. Karpinski A. C. (2019). Grit, self-efficacy, achievement orientation goals, and academic performance in university students. *Int. J. Adolesc. Youth* 25 519–535.

[B3] Ashenfelter O. Rouse C. E. (1998). *Schooling, Intelligence, and Income in America: cracks in the Bell Curve.* United States: National Bureau of Economic Research.

[B4] Azila-Gbettor E. M. Atatsi E. A. Danku L. S. Soglo N. Y. (2015). Stress and academic achievement: empirical evidence of business students in a Ghanaian polytechnic. *Int. J. Res. Bus. Stud. Manag.* 2 78–98.

[B5] Barkur R. R. Govindan S. Kamath A. (2013). Correlation between academic achievement goal orientation and the performance of Malaysian students in an Indian medical school. *Educ. Health Change Lear. Pract.* 26 98–102. 10.4103/1357-6283.120701 24200730

[B6] Barron K. E. Harackiewicz J. M. (2001). Achievement goals and optimal motivation: testing multiple goal models. *J. Pers. Soc. Psychol.* 80 706–722. 10.1037/0022-3514.80.5.706 11374744

[B7] Bergh D. Giota J. (2020). *Student Achievement Goals and Psychosomatic Health Complaints Among Swedish Adolescents: the Role of Sex.* Germany: Springer.

[B8] Chan K. W. Wong A. K. Y. Lo E. S. C. (2012). Relational Analysis of Intrinsic Motivation, Achievement Goals, Learning Strategies and Academic Achievement for Hong Kong Secondary Students. *Asia Pac. Educ. Res.* 21 230–243.

[B9] Chang T. Z. Vowles N. (2013). Strategies for improving data reliability for online surveys: a case study. *Int. J. Electron. Commer. Stud.* 4 121–129.

[B10] Chen Y. Liu X. Yan N. Jia W. Ma L. (2020). Higher Academic Stress Was Associated with Increased Risk of Overweight and Obesity among College Students in China. *Int. J. Environ. Res. Public Health* 17:5559. 10.3390/ijerph17155559 32752122 PMC7432099

[B11] Dan W. (2018). *Study on The Relations of Academic Passion and Achievement Goal Orientation and Academic Achievement of College Students.* China: Nanchang University.

[B12] Daniels L. M. Haynes T. L. Stupnisky R. H. Perry R. P. Newall N. E. Pekrun R. (2008). Individual differences in achievement goals: a longitudinal study of cognitive, emotional, and achievement outcomes. *Contemp. Educ. Psychol.* 33 584–608. 10.1016/j.cedpsych.2007.08.002

[B13] Eccles J. S. Midgley C. (1989). “Stage-environment fit: developmentally appropriate classrooms for young adolescents” in *Research on Motivation in Education.* eds Ames C. Ames R. (New York: Academic Press). 139–186.

[B14] Elliot A. J. Harackiewicz J. M. (1996). Approach and avoidance achievement goals and intrinsic motivation: a mediational analysis. *J. Pers. Soc. Psychol.* 70:461. 10.1037/0022-3514.70.3.461 8014838

[B15] Francisco P. Vera M. Lourdes M. Cristina S. Joana P. Almeida L. S. (2016). To be or not to be Retained …That’s the Question!” Retention, Self-esteem, Self-concept, Achievement Goals, and Grades. *Front. Psychol.* 7:1550. 10.3389/fpsyg.2016.01550 27790167 PMC5062915

[B16] Giota. (2006). Why am I in School? Relationships Between Adolescents’ Goal Orientation, Academic Achievement and Self-Evaluation. *Scand. J. Educ. Res.* 50 441–461. 10.1080/00313830600823803

[B17] Gonçalves T. Niemivirta M. Lemos M. S. (2017). Identification of students’ multiple achievement and social goal profiles and analysis of their stability and adaptability. *Lear. Ind. Diff.* 54 149–159. 10.1016/j.lindif.2017.01.019

[B18] Grant H. Dweck C. S. (2003). Clarifying Achievement Goals and Their Impact. *J. Pers. Soc. Psychol.* 85 541–553. 10.1037/0022-3514.85.3.541 14498789

[B19] Hall M. Hanna L. A. Hanna A. Hall K. (2015). Associations between Achievement Goal Orientations and Academic Performance Among Students at a U.K. Pharmacy School. *Am. J. Pharm. Educ.* 79 64–64. 10.5688/ajpe79564 26396273 PMC4571049

[B20] Harackiewicz J. M. Sansone C. (1991). Goals and intrinsic motivation: you can get there from here. *Adv. Motiv. Achiev.* 7 21–49.

[B21] Hong L. Lin C. D. Bray M. A. Kehle T. J. (2010). The measurement of stressful events in Chinese college students. *Psychol. Sch.* 42 315–323. 10.1002/pits.20082

[B22] Hui L. Dandan P. Guangcheng L. Jianren Z. (2018). Work Values and Organizational Commitment in Grass-roots Civil Servants: the Moderating of the Gender. *Chin. J. Clin. Psychol.* 26 561–564.

[B23] Jiali F. Miaomiao H. Yajun X. (2019). Relationships among suicide attitude and resiliency in medical students. *J. Gannan Med. Univer.* 60:636.

[B24] Jing W. Yu T. (2015). Development of the College Students’ Learning Subjective Well-being Questionnaire. *Psychol. Res.* 8 77–81.

[B25] Kaplan A. Maehr M. L. (1999). Achievement goals and student well-being. *Contemp. Educ. Psychol.* 24 330–358. 10.1006/ceps.1999.0993 10508531

[B26] Kim E. K. Furlong M. J. Ng Z. J. Huebner E. S. (2016). *Child Well-being and Children’s Rights: balancing Positive and Negative Indicators in Assessments.* Germany: Springer.

[B27] Kim K. I Won H. Liu X. Liu P. Kitanishi K. (1997). Students’ stress in China, Japan, and Korea: a transcultural study. *Int. J. Soc. Psychiat.* 43 87–94. 10.1177/002076409704300202 9252822

[B28] King R. B. Mcinerney D. M. Watkins D. A. (2012). Competitiveness is not that bad…at least in the East: testing the hierarchical model of achievement motivation in the Asian setting. *Int. J. Intercult. Relat.* 36 446–457. 10.1016/j.ijintrel.2011.10.003

[B29] King R. B. Mendoza N. B. (2020). Achievement goal contagion: mastery and performance goals spread among classmates. *Soc. Psychol. Educ.* 23 795–814. 10.1007/s11218-020-09559-x

[B30] Korpershoek H. Kuyper H. Werf G. V. (2015). Differences in students’ school motivation: a latent class modelling approach. *Soc. Psychol. Educ.* 18 137–163. 10.1007/s11218-014-9274-6

[B31] Lew B. Huen J. Yu P. Yuan L. Wang D. F. Ping F. (2019). Associations between depression, anxiety, stress, hopelessness, subjective well-being, coping styles and suicide in Chinese university students. *PLoS One* 14:e0217372. 10.1371/journal.pone.0217372 31260454 PMC6602174

[B32] Li X. Lv S. Liu L. Chen R. Zhao J. (2020). Covid-19 in guangdong: immediate perceptions and psychological impact on 304,167 college students. *Front. Psychol.* 11:2024. 10.3389/fpsyg.2020.02024 32903445 PMC7434974

[B33] Liu X. Kurita H. Uchiyama M. Ma D. (2000). Life events, locus of control, and behavioral problems among Chinese adolescents. *J. Clin. Psychol.* 56:1565. 10.1002/1097-4679(200012)56:12<1565::aid-7>3.0.co;2-u 11132571

[B34] Liu X. Tein J. Y. (2005). Life events, psychopathology, and suicidal behavior in Chinese adolescents. *J. Affect. Disord.* 86 195–203. 10.1016/j.jad.2005.01.016 15935239

[B35] Luo W. Paris S. G. Hogan D. Luo Z. (2011). Do performance goals promote learning? A pattern analysis of Singapore students’ achievement goals. *Contemp. Educ. Psychol.* 36 165–176. 10.1016/j.cedpsych.2011.02.003

[B36] Mahdy M. (2020). The Impact of COVID-19 Pandemic on the Academic Performance of Veterinary Medical Students. *Front. Vet. Sci.* 7:594261. 10.3389/fvets.2020.594261 33134368 PMC7572855

[B37] Marques G. Drissi N. Díez I. Abajo B. Ouhbi S. (2021). Impact of COVID-19 on the psychological health of university students in Spain and their attitudes toward Mobile mental health solutions. *Int. J. Med. Inform.* 147:104369. 10.1016/j.ijmedinf.2020.104369 33388481 PMC9759811

[B38] Nicholls J. G. (1984). Achievement motivation: conceptions of ability, subjective experience, task choice, and performance. *Psychol. Rev.* 91 328–346. 10.1037/0033-295x.91.3.328

[B39] Noddings N. (2010). Happiness and Education. *J. Philos. Educ.* 2 17–29.

[B40] Pekrun R. Elliot A. J. Maier M. A. (2006). Achievement goals and discrete achievement emotions: a theoretical model and prospective test. *J. Educ. Psychol.* 98 583–597. 10.1037/0022-0663.98.3.583

[B41] Pollard E. L. Lee P. D. (2003). Child Well-being: a Systematic Review of the Literature. *Soc. Indicat. Res.* 61 59–78. 10.1023/A:1021284215801

[B42] Schütz F. F. Bedin L. M. Sarriera J. C. (2018). Subjective Well-Being of Brazilian Children from Different Family Settings. *Appl. Res. Qual. Life* 14 1–14.

[B43] Schwinger M. Steinmayr R. Spinath B. (2016). Achievement Goal Profiles in Elementary School: antecedents, Consequences, and Longitudinal Trajectories. *Contemp. Educ. Psychol.* 46 164–179. 10.1016/j.cedpsych.2016.05.006

[B44] Shi Y. Zhang S. E. Fan L. Sun T. (2021). What motivates medical students to engage in volunteer behavior during the covid-19 outbreak? a large cross-sectional survey. *Front. Psychol.* 11:569765. 10.3389/fpsyg.2020.569765 33519583 PMC7844061

[B45] SideridiS G. D. (2005). Goal Orientation, Academic Achievement, and Depression: evidence in Favor of a Revised Goal Theory Framework. *J. Educ. Psychol.* 97 366–375. 10.1037/0022-0663.97.3.366

[B46] Sideridis G. D. (2020). A physiological analysis of achievement goal orientations under pressure: an experimental analysis. *Int. J. Sch. Educ. Psychol.* 8 1–12.

[B47] SkaalviK E. M. (1997). Self-enhancing and self-defeating ego orientation: relations with task and avoidance orientation, achievement, self-perceptions, and anxiety. *J. Educ. Psychol.* 89 71–81. 10.1037/0022-0663.89.1.71

[B48] Stinebrickner R. Stinebrickner T. R. (2007). The Causal Effect of Studying on Academic Performance. *Nephron Clin. Pract.* 8 1868–1868.

[B49] Strelhow M. Sarriera J. C. Casas F. (2020). Evaluation of Well-Being in Adolescence: proposal of an Integrative Model with Hedonic and Eudemonic Aspects. *Child Indic. Res.* 13 1–14.

[B50] Swetlik C. Franco K. N. (2018). *Medical Student Suicide: an Assessment of Risk Factors and Prevention Strategies.* Germany: Springer.

[B51] Tapola A. Jaakkola T. Niemivirta M. (2014). The Influence of Achievement Goal Orientations and Task Concreteness on Situational Interest. *J. Exp. Educ.* 82 455–479. 10.1080/00220973.2013.813370

[B52] Tapola A. Niemivirta M. (2008). The role of achievement goal orientations in students” perceptions of and preferences for classroom environment. *Br. J. Educ. Psychol.* 78 291–312. 10.1348/000709907x205272 17535519

[B53] Tian L. Yu T. Scott H. E. (2017). Achievement Goal Orientations and Adolescents’ Subjective Well-Being in School: the Mediating Roles of Academic Social Comparison Directions. *Front. Psychol.* 8:37. 10.3389/fpsyg.2017.00037 28197109 PMC5281619

[B54] Tjin A. T. S. L. N. M. de Boer A. Croiset G. Koster A. S. van der Burgt S. Kusurkar R. A. (2018). How basic psychological needs and motivation affect vitality and lifelong learning adaptability of pharmacists: a structural equation model. *Adv. Health Sci. Educ.* 23 549–566. 10.1007/s10459-018-9812-7 29388088

[B55] Tuominen H. Niemivirta M. Lonka K. Salmela-Aro K. (2020). Motivation across a transition: changes in achievement goal orientations and academic well-being from elementary to secondary school. *Lear. Ind. Diff.* 79:101854. 10.1016/j.lindif.2020.101854

[B56] Tuominen-Soini H. Salmela-Aro K. Niemivirta M. (2008). Achievement goal orientations and subjective well-being: a person-centred analysis. *Lear. Instruct.* 18 251–266. 10.1016/j.learninstruc.2007.05.003

[B57] Tuominen-Soini H. Salmela-Aro K. Niemivirta M. (2011). Stability and change in achievement goal orientations: a person-centered approach. *Contemp. Educ. Psychol.* 36 82–100. 10.1016/j.cedpsych.2010.08.002

[B58] Tuominen-Soini H. Salmela-Aro K. Niemivirta M. (2012). Achievement goal orientations and academic well-being across the transition to upper secondary education. *Learn. Ind. Diff.* 22 290–305. 10.1016/j.lindif.2012.01.002

[B59] Weina N. (2019). *The Influence of Achievement Goal Orientation on Student Cyberloafing and its Psychological Mechansim.* China: Central China Normal University.

[B60] Werneck A. O. Silva D. R. Malta D. C. Lima M. G. Souza-Jú P. R. B. Azevedo L. O. (2020). The mediation role of sleep quality in the association of the incidence of unhealthy movement behaviors due to COVID-19 quarantine and mental health. *Sleep Med.* 76 10–15. 10.1016/j.sleep.2020.09.021 33049547 PMC7518797

[B61] Xiaozhou Z. Ting P. Jie L. (2019). Relationship between Achievement Goal Orientation and Perceived Social Support among High School Students: the Moderation Effect of Gender. *J. Guizhou Normal Univer.* 37 116–122.

[B62] Xue Y. Haimiao C. Shuiping H. (2018). Relationship between the Achievement Goal Orientation and Time Management Disposition of medical college students. *J. Diet Health* 5 279–280.

[B63] Xue Z. (2019). *Research on Time Management Disposition of College Students Based on Psychological Control Sources and Achievement Goal Orientation.* China: Liaoning Normal University.

[B64] Yanfei W. Yunjian L. Yuexin H. (2011). A Study on the Relationship between Psychological capital achievement goal Orientation and Academic Achievement of College Students. *High. Educ. Explor.* 6 128–136.

[B65] Yang W. Zhou W. (2008). What Accounts for Chinese-American Children’s High Academic Performance: a Literature Review Of Parental Influences And Home Environment. *Gift. Educ. Int.* 24 88–104. 10.1177/026142940802400111

[B66] You J. W. (2021). Investigating the effects of achievement goals on team creativity and team achievement in learning communities at a South Korean university. *High. Educ.* 81 367–383. 10.1007/s10734-020-00545-y

[B67] Youhao Y. Yulin G. Ting L. (2019). The mediating role of subjective well-being in the relationship between the sense of life meaning and learning motivation among medical students. *Chin. Nurs. Res.* 33 1482–1485.

[B68] Yuting X. Yifei C. Shuang Z. (2020). Relationship among academic achievement, learning happiness, values of learning, and learning attitudes among undergraduate nursing students. *J. Nurs. Sci.* 35 68–70.

[B69] Zang S. Y. Carrasquillo A. L. (1995). Chinese parents’ influence on academic performance. *N. Y. State Assoc. Biling. Educ. J.* 10 46–53.

[B70] Zhang S. E. Liu W. Wang J. Shi Y. Fan L. (2018). Impact of workplace violence and compassionate behaviour in hospitals on stress, sleep quality and subjective health status among chinese nurses: a cross-sectional survey. *BMJ Open* 8:e019373. 10.1136/bmjopen-2017-019373 30287664 PMC6194400

[B71] Zhang Y. Watermann R. Daniel A. (2016). Are multiple goals in elementary students beneficial for their school achievement? A latent class analysis. *Lear. Ind. Diff.* 51 100–110. 10.1016/j.lindif.2016.08.023

[B72] Zhou X. Liao X. Spiegelman D. (2017). “cross-sectional” stepped wedge designs always reduce the required sample size when there is no time effect. *J. Clin. Epidemiol.* 83 108–109. 10.1016/j.jclinepi.2016.12.011 28093263 PMC5517056

